# Neoepitope fragments as biomarkers for different phenotypes of intervertebral disc degeneration

**DOI:** 10.1002/jsp2.1215

**Published:** 2022-07-06

**Authors:** Shangbin Cui, Wenyue Li, Graciosa Q. Teixeira, Cornelia Neidlinger‐Wilke, Hans‐Joachim Wilke, Lisbet Haglund, Hongwei Ouyang, R. Geoff Richards, Sibylle Grad, Mauro Alini, Zhen Li

**Affiliations:** ^1^ AO Research Institute Davos Davos Switzerland; ^2^ Guangdong Provincial Key Laboratory of Orthopedics and Traumatology The First Affiliated Hospital of Sun Yat‐Sen University Guangzhou China; ^3^ Zhejiang University‐University of Edinburgh Institute (ZJU‐UoE Institute) Zhejiang University Haining China; ^4^ Institute of Orthopedic Research and Biomechanics, Centre for Trauma Research Ulm (ZTF Ulm) Ulm University Ulm Germany; ^5^ Department of Surgery and Shriners Hospital for Children McGill University Montreal Canada

**Keywords:** biomarker, bioreactor, intervertebral disc degeneration, neoepitope, organ culture model

## Abstract

**Background:**

During the intervertebral disc (IVD) degeneration process, initial degenerative events occur at the extracellular matrix level, with the appearance of neoepitope peptides formed by the cleavage of aggrecan and collagen. This study aims to elucidate the spatial and temporal alterations of aggrecan and collagen neoepitope level during IVD degeneration.

**Methods:**

Bovine caudal IVDs were cultured under four different conditions to mimic different degenerative situations. Samples cultured after 1‐ or 8‐days were collected for analysis. Human IVD samples were obtained from patients diagnosed with lumbar disc herniation (LDH) or adolescent idiopathic scoliosis (AIS). After immunohistochemical (IHC) staining of Aggrecanase Cleaved C‐terminus Aggrecan Neoepitope (NB100), MMP Cleaved C‐terminus Aggrecan Neoepitope (MMPCC), Collagen Type 1α1 1/4 fragment (C1α1) and Collagenase Cleaved Type I and II Collagen Neoepitope (C1,2C), staining optical density (OD)/area in extracellular matrix (OECM) and pericellular zone (OPCZ) were analyzed. Conditioned media of the bovine IVD was collected to measure protein level of inflammatory cytokines and C1,2C.

**Results:**

For the bovine IVD sections, the aggrecan MMPCC neoepitope was accumulated in nucleus pulposus (NP) and cartilage endplate (EP) regions following mechanical overload in the one strike model after long‐term culture; as for the TNF‐α induced degeneration, the OECM and OPCZ of collagen C1,2C neoepitope was significantly increased in the outer AF region after long‐term culture; moreover, the C1,2C was only detected in conditioned medium from TNF‐α injection + Degenerative loading group after 8 days of culture. LDH patients showed higher MMPCC OECM in NP and higher C1,2C OECM in AF region compared with AIS patients.

**Conclusions:**

In summary, aggrecan and collagen neoepitope profiles showed degeneration induction trigger‐ and region‐specific differences in the IVD organ culture models. Different IVD degeneration types are correlated with specific neoepitope expression profiles. These neoepitopes may be helpful as biomarkers of ECM degradation in early IVD degeneration and indicators of different degeneration phenotypes.

## Graphical abstract

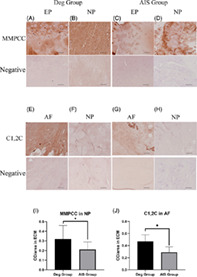

## 1 INTRODUCTION

Low back pain (LBP) is responsible for 60.1 million disability adjusted life‐years. There was a 54% increase of incidence since 1990, with the biggest increase seen in low‐income and middle‐income countries.^1^ According to the latest data from the Global Burden of Disease Project published in 2017, the global prevalence of LBP was 7.8%, meaning that 577 million people are affected.^2^, ^3^ Intervertebral disc (IVD) degeneration is the most common reason for LBP.^4^, ^5^

The IVD has a heterogeneous structure with three distinct regions: the central nucleus pulposus (NP), the outer annulus fibrosus (AF) and the cartilage endplate (CEP). The major biochemical components of the extracellular matrix (ECM) in mature IVD tissue are collagen, proteoglycan, elastin, and glycoproteins. Collagen comprises approximately 70% of the dry weight of the mature disc, with collagen type I decreasing in expression from the AF to the NP, and collagen type II increasing in expression from AF to the NP.^6^ There are two types of proteoglycan (PG) molecules in the disc that can be subdivided into two distinct classed, aggregating and non‐aggregating PGs, depending upon the ability to interact with hyaluronan. Accumulating evidence indicates that a significant component of the non‐aggregating PG content of the disc is a cumulative turnover product of large aggregating PGs.^7^, ^8^ In general, the glycosaminoglycan (GAG) content of the disc is greatest within the NP, decreasing toward the edges of the AF.^9^ Aggrecan is the primary PG responsible for the water‐retaining properties of the disc, due to its attached sulfated GAG chains.^10^ During IVD degeneration, the ECM of the IVD becomes highly disorganized and denatured.^11^, ^12^ There are noticeable differences between the under‐50 and the over‐50‐year‐old group regarding both collagen and proteoglycan (PG) content of the IVD.^13^ In the initial stage of degeneration, collagen synthesis, in general, increases, with a clear increase in type II collagen in the NP, presumably indicating the presence of a repair mechanism.^14^ With the progress of degeneration, the synthesis pattern changes and more type II collagen is synthesized in the outer annulus fibrosus (AF), forming strong collagen fibrils; nevertheless, type I collagen is synthesized in the inner region of the AF and NP of these discs.^15^ The synthesis of PGs changes with a decline in the production of aggrecan and an increase in the production of versican, biglycan, decorin, and fibronectin,^16^ together with the modification of glycosaminoglycans side chain (GAGs) from chondroitin sulfate to keratan sulfate.

In degenerated IVD, the loss of ECM components exceeds de novo synthesis, whereas, in healthy IVD, the turnover and synthesis of ECM molecules are at equilibrium.^17^ Disc composition changes with age, leading to a state of disc degeneration resulting from biochemical, genetic inheritance, and environmental factors. Changes in collagen type and decreased PG result in a loss of tissue integrity, decreased hydration, drop of the viscoelastic properties and inability to withstand load.^18^

**FIGURE 1:**
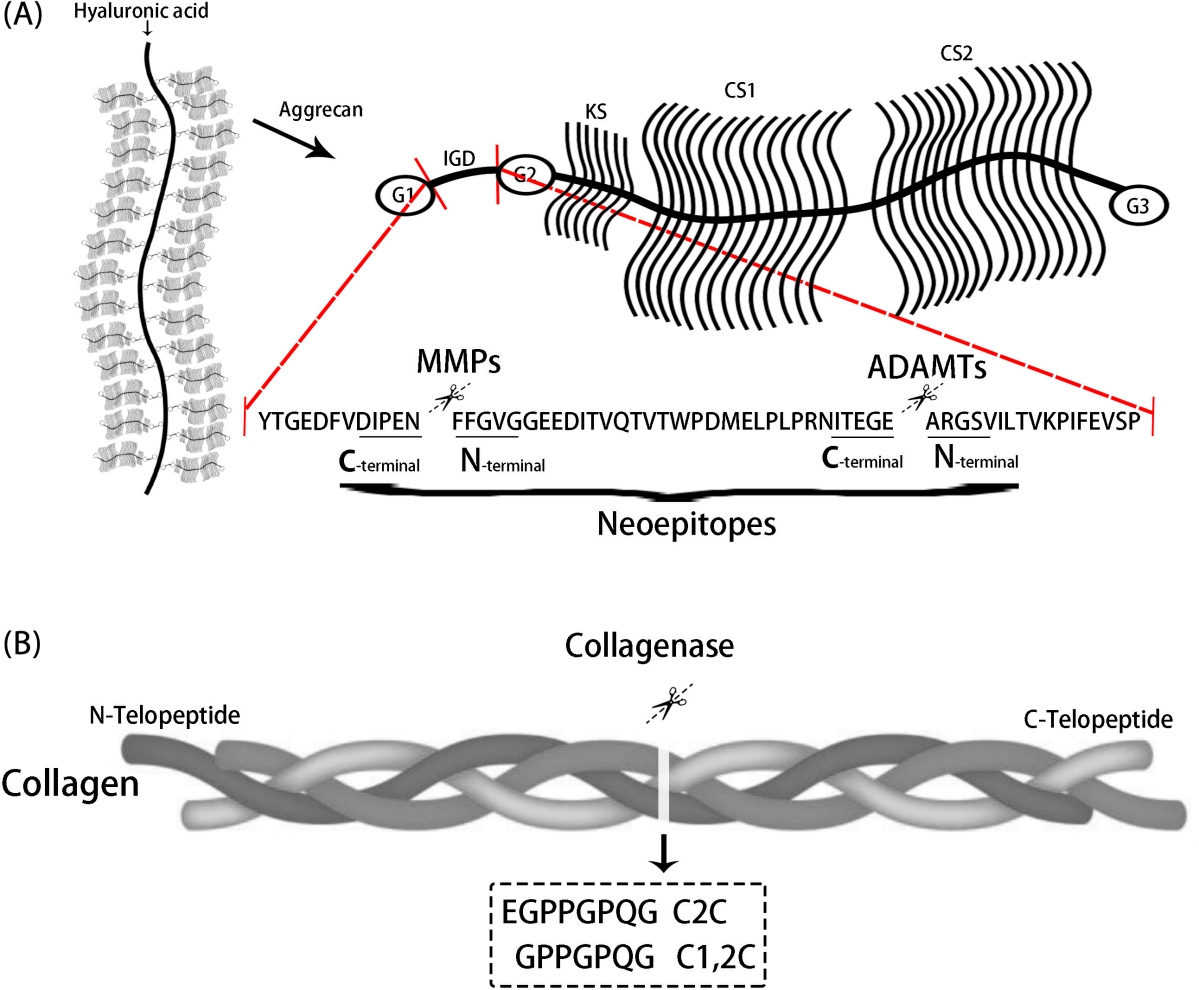
(A) The enzymatic cleavage sites of aggrecan and protein sequence of aggrecan neoepitopes. IGD, interglobular domain, KS, keratan sulfate side chains, CS, chondroitin sulfate side chains. (B) The enzymatic cleavage sites of collagen and protein sequence of collagen neoepitopes

Degradation of aggrecan and collagen in the IVD is an essential aspect of normal growth and development but it is also involved in IVD pathology.^19^ It is evident from the changes in collagen and PG macromolecular structure that proteolysis is a major contributor to the age or degeneration related changes taking place in the disc.^18^ Several families of proteinases are involved in this process including aggrecanases and matrix metalloproteinases (MMPs).^20^ Collagenases (MMP‐1, ‐8 and ‐13), gelatinases (MMP‐2 and ‐9), and stromelysin (MMP3) have been found to be present in the disc, and their actions account for the degradation of collagen, aggrecan, versican, and link protein.^21^, ^22^, ^23^, ^24^, ^25^ Aggrecan and versican degradation may also result from members of a second family of metalloproteinases, ADAMTs.^25^ Two members of this family (ADAMTS‐4 and ‐5) show a particular degradation effect on aggrecan and have been termed aggrecanases.^26^ Enzymatic cleavage at specific sites of matrix proteins leads to minimal structural damage associated with the appearance of neoepitopes during the early phase of IVD degeneration (Figure 1).^19^, ^27^, ^28^ These neoepitopes may thus predict functional failure of the IVDs. However, there have been no studies on the detailed distribution of aggrecan and collagen neoepitopes in the IVD during the process associated with normal disc matrix turnover and at the very early degeneration phase.

In recent years, bovine IVD organ culture models have gained popularity since the dimensions and biological composition of bovine IVDs is relatively equivalent to human discs.^29^ Investigating the degeneration mechanism in whole IVD explants exposed to a specific loading regime may represent a way to bridge the gap between in vitro and in vivo tests.^30^, ^31^ In this study, detrimental mechanical load and/or inflammatory cytokines were applied to bovine caudal IVDs in organ culture to simulate different scenarios of early IVD degeneration. The objective of this study was to use immunohistochemistry to reveal the differential distribution of neoepitopes formed due to the degradation of matrix components with the onset of ex vivo induced disc degeneration. In the current study, different IVD organ culture models^32^, ^33^, ^34^ were established to mimic the different phenotypes of IVD degeneration: Phys—**Phys**iological loading was applied to mimic the normal physiological compression on discs^33^; OS + Phys—**O**ne **S**trike model was established to simulate acute mechanical overload injury, a commonly seen injury type in clinical situations,^34^ Deg—**Deg**enerative loading was performed to induce disc degeneration by fatigue loading on discs^32^; and Deg + TNF‐α—**TNF‐α** intradiscal injection was applied in combination with degenerative loading to further induce an inflammatory response of the discs.^32^ Recently, it was suggested that the “first hit” (initial damaging event, i.e., trauma, or infection) in the course of intervertebral disc degeneration is accompanied by an inflammatory environment within the IVD.^35^ TNF‐α is one of the key proinflammatory cytokines contributing to disc degeneration, inflammation and discogenic pain. TNF‐α also regulates the expression of major catabolic enzymes for ECM deterioration such as ADAMTs and MMPs.^36^, ^37^, ^38^ Our previous study^32^ has shown that TNF‐α in combination with degenerative loading could induce an inflammatory response of native disc cells, as indicated by upregulated expression of IL‐1β, IL‐6, and IL‐8. Therefore, this Deg + TNF‐α model was used as an inflammatory degenerative model. The spatial and temporal expression profile of aggrecan and collagen neoepitopes were analyzed in these organ culture models. Expression of the neoepitopes was also validated in herniated human IVD and adolescent idiopathic scoliosis (AIS) samples. Matrix degradation products and proinflammatory cytokines released into the conditioned medium were measured with ELISA.

## 2 MATERIALS AND METHODS

### 2.1 Dissection and culture of bovine intervertebral discs with endplates

Caudal bovine IVDs were obtained from 4 to 10 months animals (two males, two female) as previously described.^31^, ^39^, ^40^ Briefly, after removal of the soft tissues, IVDs comprising cartilage and bony endplates (about 1 mm thick on each side) were harvested using a bandsaw and rinsed with Ringer solution using the Pulsavac jet‐lavage system (Zimmer, Warsaw, IN). The discs were further incubated in 1000 units/ml penicillin, 1000 μg/ml streptomycin in phosphate buffered saline (PBS) solution for 10 min. The discs were transferred to 6‐well plates and kept in an incubator at 37°C, 85% humidity and 5% CO_2_ until the next day. The culture medium was composed of Dulbecco's modified Eagle's medium (DMEM) containing 4.5 g/L glucose and supplied with 2% fetal calf serum, 1% ITS+ Premix (Discovery Labware, Inc., Bedford, MA), 50 μg/ml ascorbate‐2‐phosphate (Sigma–Aldrich, St. Louis, MO), non‐essential amino acids, 100 units/ml penicillin, 100 μg/ml streptomycin (all except for mentioned, Gibco, Basel, Switzerland) and 0.1% Primocin (Invitrogen, San Diego, CA). The average disc height was 9.47 ± 1.27 mm and average disc diameter was 15.96 ± 1.75 mm.

### 2.2 Loading protocol

**FIGURE 2:**
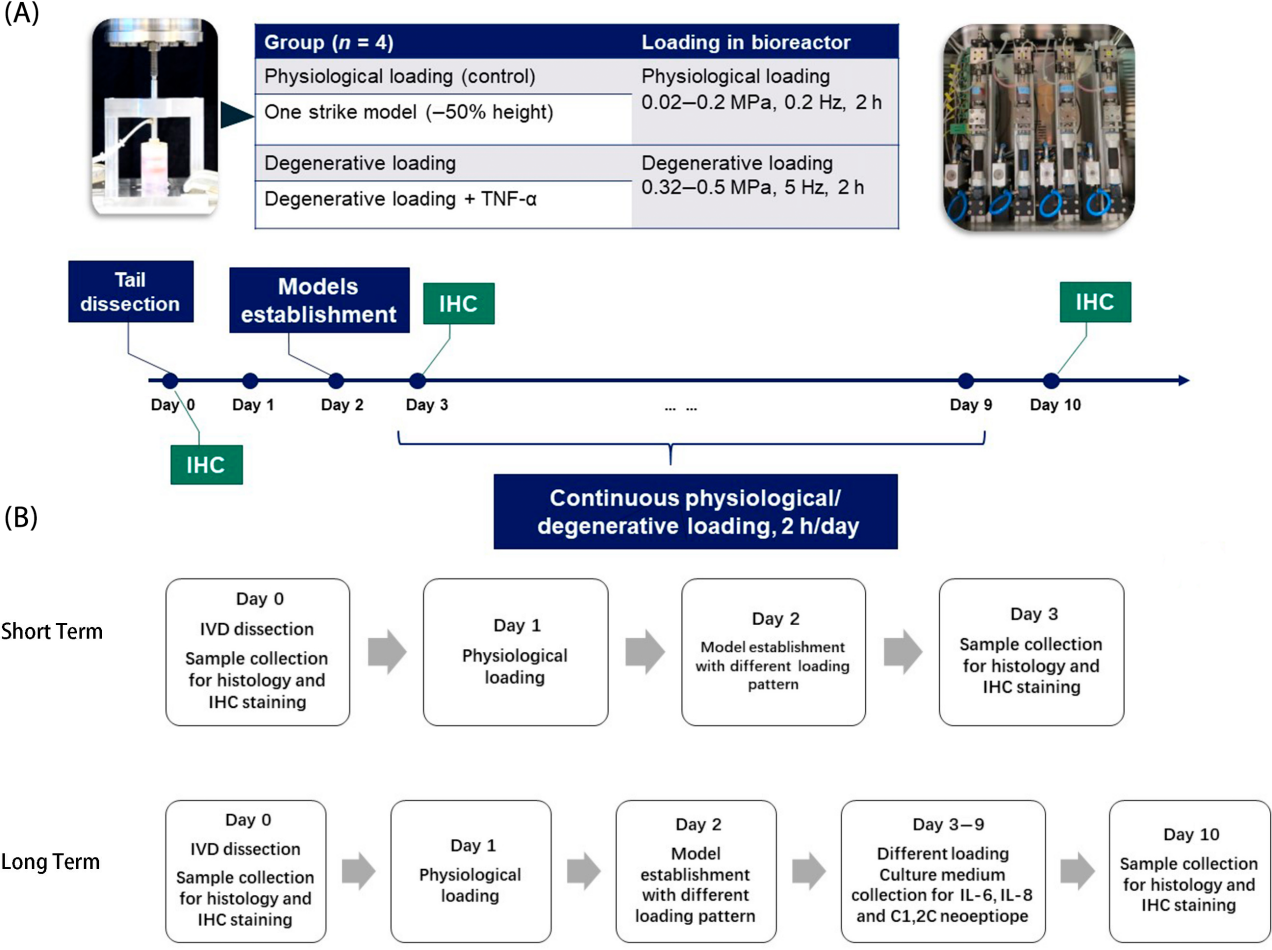
(A) The flowchart and group design of the experiment. The picture on the left shows the MTS machine with custom designed chamber for one strike loading on bovine IVDs. The picture on the right shows the bioreactor for daily physiological/degenerative loading. (B) The sample measurement flowchart of short‐term and long‐term studies

Discs were systematically assigned to one of the five groups to achieve equivalent disc size and distribution of disc levels for each group: Day 0 group; Group 1—Physiological loading group (G1: Phys); Group 2—One Strike + Physiological loading group (G2: OS + Phys); Group 3—Degenerative loading group (G3: Deg) and Group 4—TNF‐α injection + Degenerative loading group (G4: TNFα + Deg). IVDs were cultured under four different conditions (Figure 2A): (G1) Phys—Physiological loading was performed with our custom designed bioreactor at 0.02–0.2 MPa, 0.2 Hz, 2 h per day.^41^ (G2) OS + Phys—One strike loading was performed using a Mini Bionix 858 MTS machine and a custom designed chamber.^34^ IVDs were held under a mild load of 10 N for 3 min, followed by compression to 50% of disc height in 1 s. The average peak stress induced by one strike loading was 35.461 ± 8.274 MPa. (G3) Deg—Degenerative loading was performed with our custom designed bioreactor at 0.32–0.50 MPa, 5 Hz, 2 h per day.^32^ (G4) Deg + TNF‐α—After the first cycle of degenerative loading, recombinant human TNF‐α (R&D systems, Zug, CH) was injected with a 30‐gauge insulin needle (Braun, Melsungen, GE) into the NP tissue at a concentration of 100 ng TNF‐α within 70 μl PBS/IVD.^32^, ^34^

The bioreactor was maintained in an incubator at 37°C, 85% humidity and 5% CO_2_. After daily loading, the IVDs were cultured in 6‐well plates for overnight free swelling recovery. The medium of the IVD samples was changed twice a day, after loading and after free swelling. After model induction (OS load in G2; Deg loading in G3; Deg loading and TNF‐α injection in G4) on Day 2, the IVDs were cultured under physiological loading (G1—Phys and G2—OS + Phys) or degenerative loading (G3—Deg and G4—TNF‐α + Deg) for 1 or 8 days (Figure 2).

### 2.3 Sample preparation for histology and immunohistochemistry

IVD samples were collected on Day 0, Day 3, and Day 10. To maximally reveal the neoepitope expression in the disc, a high‐speed drill (K.5250 Woodcarving Kit, Foredom Electric Co.) was used to remove all the bony endplate and avoid the decalcification procedure. Samples were fixed with formalin for 3 days. After fixation, the samples were washed with deionized water thoroughly and with 50% ethanol for 2 days on a shaker. The samples were then stored in 70% ethanol and followed by paraffin embedding. Sagittal sections of IVDs were cut at a thickness of 8 μm using a microtome (Microm, Germany).

### 2.4 Safranin‐O Fast Green staining

Paraffin sections were dewaxed and rehydrated. In the deparaffinization procedure, sections were put on a hot plate (60°C) for 3 min until the paraffin was melted. Then the sections were washed with xylene twice for 4 min. After deparaffinizing, sections were rehydrated with descending concentrations of ethanol (100% 2 min × 2 times, 96% 2 min, 70% 2 min and 50% 2 min) and deionized water 2 min. Sections were then stained with 0.1% Safranin‐O (Chroma Gesellschaft, Munster, Germany) and 0.02% Fast Green (Fluka, Seelze, Germany) to reveal proteoglycan and collagen deposition respectively and counterstained with Weigert's hematoxylin to reveal cell distribution. After staining, sections were dehydrated with ascending concentrations of ethanol (50%, 70%, 96%, and 100%) and xylene, then coversliped with Eukitt (Fluka). The sections were imaged in transmitted light under an upright optical microscope (Olympus BX63, Japan).

### 2.5 Immunohistochemistry

The detailed immunohistochemistry protocol is supplied in the supplementary information. Paraffin sections were dewaxed and rehydrated as described above. Endogenous peroxidase activity was blocked by incubation with 0.3% hydrogen peroxide for 30 min. Afterwards, heterogenetic antigen was blocked with goat serum (# S‐1000, Vector Laboratories, Burlingame, CA, 1:20 dilution in PBS‐Tween) for 1 h at room temperature.

Samples were incubated with primary antibody at 4°C overnight. Antibodies and their concentrations used for detection of different neoepitopes are shown in Table 1. The sections were washed with PBS‐Tween, and then incubated with secondary antibody (Biotinylated Anti‐Rabbit IgG[H + L] made in goat, CA#BA‐1000, Vector Laboratories, Burlingame) at room temperature for 30 min. After washing with PBS‐Tween, the sections were incubated with ABC‐complex (#PK‐6100, Vector Laboratories) at room temperature for 30 min. Following thorough washing with PBS‐Tween, the sections were incubated with DAB (3,3′‐diaminobenzidine) for 5 min, until color was detected, followed by counterstaining with hematoxylin. The sections were washed, dehydrated using ascending concentrations of ethanol (50%, 70%, 96%, 100%) and xylene. Then the sections were mounted with Eukitt. Negative control sections were incubated with PBS‐Tween and goat serum without the primary antibody, and the same secondary IgG antibody.

**TABLE 1:** Aggrecan and collagen neoepitope antibodies used for IHC.

Neoepitope	Antibody	Company/source	Specificity	Working concentration
NB100	Aggrecanase Cleaved C‐terminus Aggrecan Neoepitope Antibody	NB100‐74350, Novus	NITEGE	1 μg/ml
MMPCC	MMP Cleaved C‐terminus Aggrecan Neoepitope (MMPCC) Antibody	#1319, kindly supplied by Lisbet Haglund, Montreal, Canada	SYN 78 (CGGFVDIPEN)	1:500 dilution in PBS‐Tween
C1α1	Collagen Type 1α1, 1/4 Fragment (C1α1) Antibody	#3277, kindly supplied by Lisbet Haglund, Montreal, Canada	SYN 997 (AGQRGGC)	1:500 dilution in PBS‐Tween
C1,2C	Collagenase (MMPs) Cleaved Type I and II Collagen Neoepitope (C1,2C) Antibody	C1,2C Polyclonal Rabbit AB, 20‐1035, 100 μl/vial Rabbit serum, IBEX	Α‐chain fragments containing an 8 amino acid sequence at the C terminus of the 1/4 fragment	1:500 dilution in PBS‐Tween

The immunoreaction was repeated on four biological replicates per group for each neoepitope. All the samples were processed in parallel to maintain the same experiment condition.

### 2.6 IHC staining of human IVD sections

Human IVD tissue was collected from patients diagnosed with lumbar disc herniation (LDH, *n* = 3, 2 females and 1 male, age 45.3 ± 7.3 years) or adolescent idiopathic scoliosis (AIS, *n* = 3, 2 females and 1 male, age 16.7 ± 3.1 years). Informed consent for sample collection was obtained from each patient and the study was approved by the ethical committee of Ulm University (number: 371/19). The IVD samples were embedded in paraffin and sections with 5 μm thickness were made. The sections were stained with MMPCC and C1,2C antibodies as described above. The immunoreaction was repeated with three biological replicates per group for each neoepitope. All the samples were processed in parallel to maintain the same staining condition.

### 2.7 IHC image analysis

**FIGURE 3:**
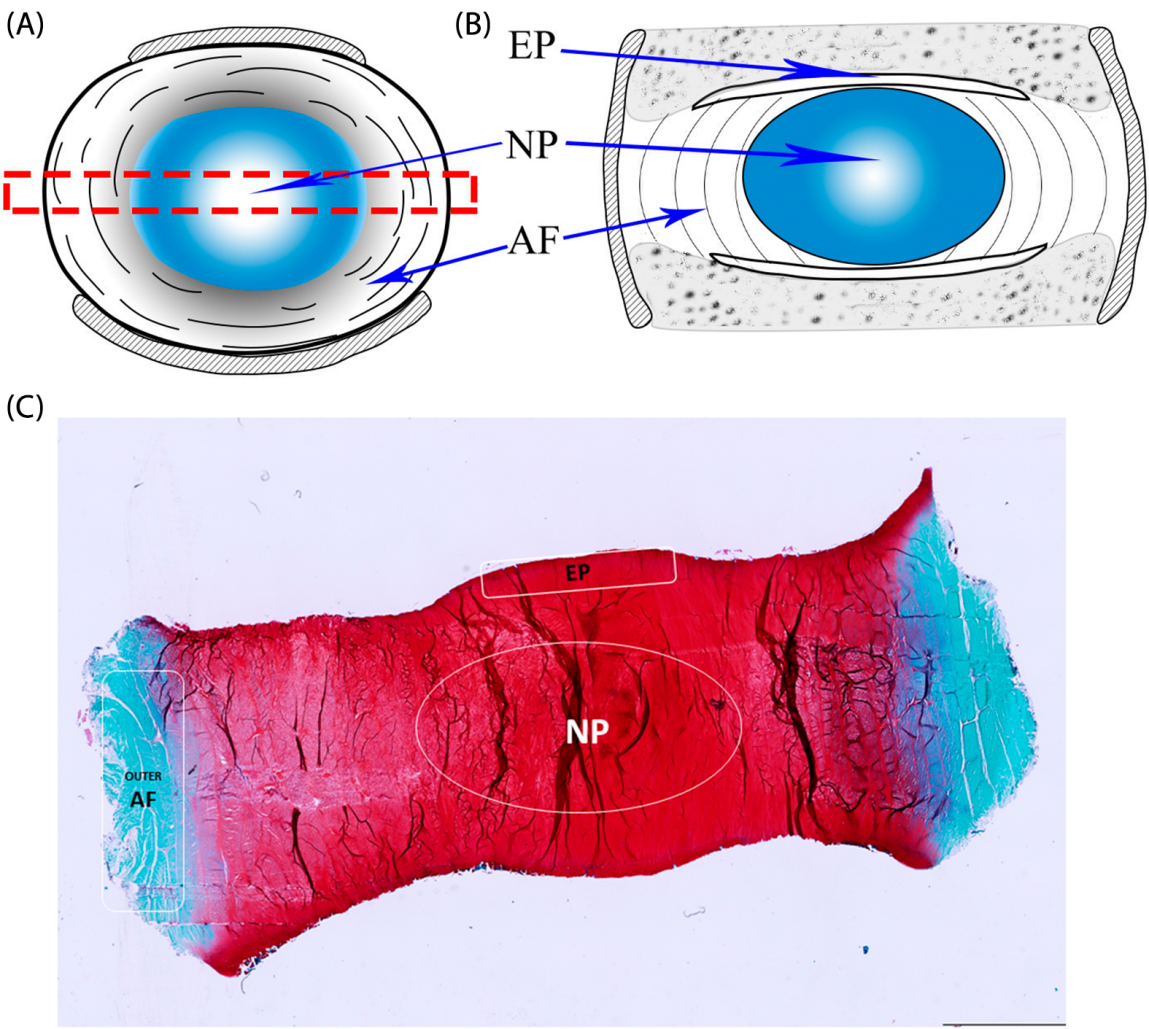
(A) Schematic drawing of IVD cross section. The red box represents the medial region of the IVD that was used to make the sagittal sections. (B) Schematic drawing of IVD sagittal section. (C) Representative Safranin‐O Fast Green stained image of IVD sagittal section labeled with regions of interest (ROI). The ROI include nucleus pulposus (NP), outer annulus fibrosus (AF) and the middle of cartilage endplate (EP). Scale bar 5 mm

For analysis, each bovine disc was morphologically separated into three areas, nucleus pulposus (NP), outer annulus fibrosus (AF), and cartilage endplate (EP) (Figure 3). Four IVD sections for each group, and three images per region of interest for each section randomly selected were used for semi‐quantitative analysis. All assessors were blinded to study groups. The intensity of DAB staining in pericellular zone,^34^ and in the extracellular matrix was evaluated semi‐quantitatively by Optical Density (OD)/Area method with IMAGEPRO PLUS 7 software (Media Cybernetics, Inc.). The OD/Area in **E**xtra**c**ellular **M**atrix was named as OECM, the OD/Area in **P**eri**c**ellular **Z**one was named as OPCZ^34^, ^42^ (Supplementary Figure 5B). As for the human IVD samples, the OECM of NP, AF, and EP regions were analyzed in six sections (two sections from each donor) from each group, with three images per region of interest for each section.

### 2.8 Analysis of conditioned medium

Conditioned media with a volume of 5 ml during loading and 7 ml during free swelling per disc was collected for analysis of released inflammatory cytokines and matrix components. IL‐6 and IL‐8 protein levels were measured with bovine IL6 and IL‐8 ELISA kits (Kingfisher Biotech, St. Paul, MN). The C1,2C epitope was measured with Collagen Type I and II Cleavage Assay ELISA kit (IBEX, Montreal, Quebec, Canada).

### 2.9 Statistical analysis

Statistical analysis was performed using GraphPad Prism 7 software (GraphPad Software, Inc., La Jolla, CA). Non‐parametric tests were performed for all the analyze. Kruskal‐Wallis test was used for multiple comparison with Tukey's post hoc test. *p <* 0.05 was considered statistically significant.

## 3 RESULTS

### 3.1 Safranin‐O Fast Green staining and IHC staining overview

**FIGURE 4:**
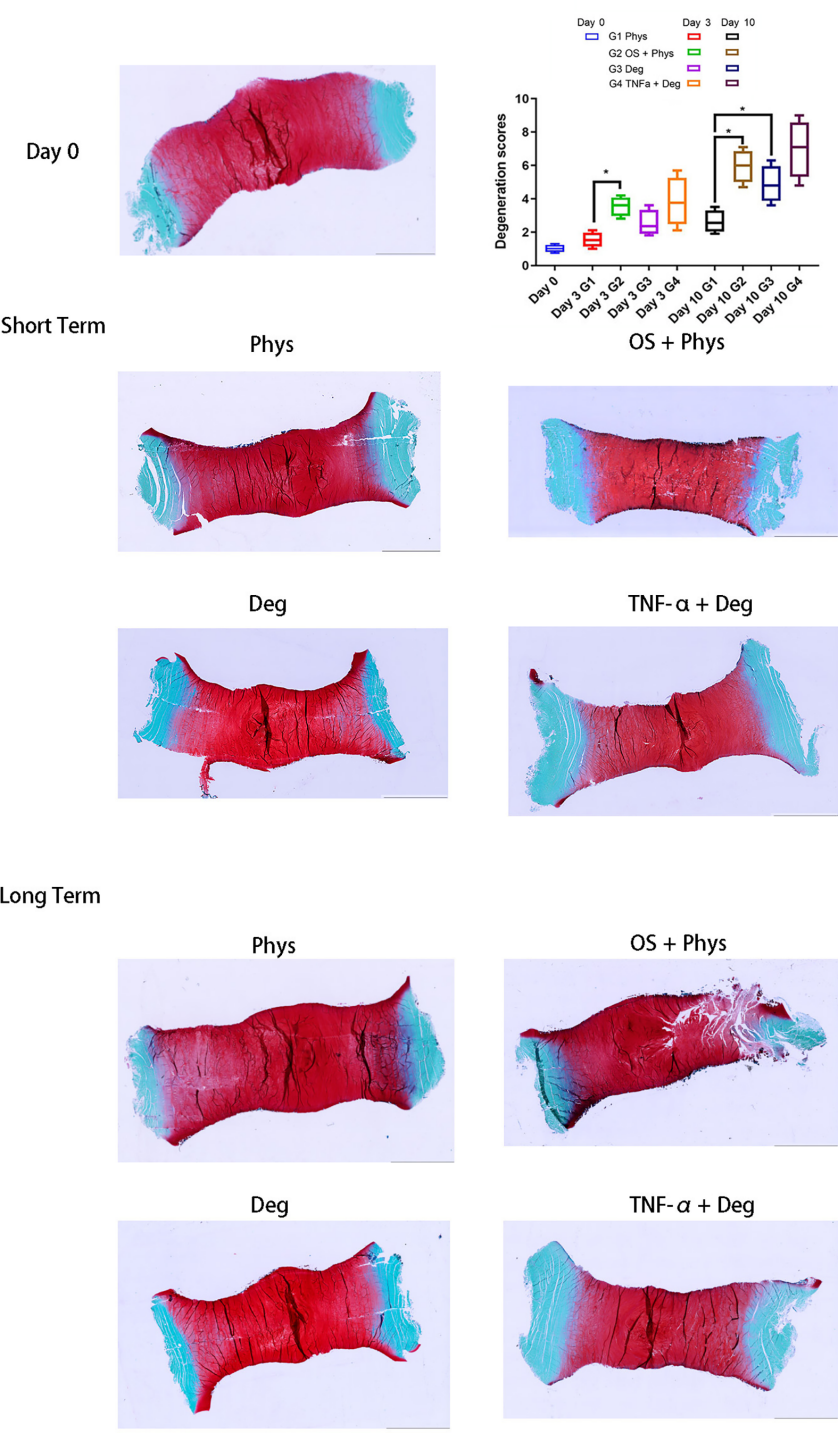
Sagittal IVD sections stained with Safranin‐O Fast Green. Day 0 group was from the IVD right after dissection; Phys, OS + Phys, Deg, TNF‐α + Deg groups were from the IVD after short‐term and long‐term loading. Scale bar 5 mm. The degeneration scores are shown on the top right corner. *N* = 4, median with interquartile range, *p < 0.05

Safranin O Fast Green stained images from Day 0, short‐term and long‐term studies are shown in Figure 4. The OS + Phys group showed fissures through the EP into the AF and NP regions. The microstructure of IVD within this group was damaged because of the high impact one strike load. A semi‐quantitative scoring scheme described in our previous study^34^ was used to evaluate the Safranin O/Fast Green staining. The IVD structure/cleft characteristics were summed up to assess the degree of IVD degeneration. Six regions of interest were randomly selected from each section. There was significant difference between OS + Phys and Phys groups on both Day 3 and Day 7 (*p* < 0.05). Deg group showed higher degenerative scores compared with Phys group on Day 7 (*p* < 0.05).

The whole IVD overview IHC images of all the 4 neoepitopes are shown in Supplementary Figures 1–4. Compared with other neoepitopes, NB100 showed weakest staining intensity in NP, AF and EP regions (Supplementary Figure 1). MMPCC was expressed in NP, AF and EP regions and the staining intensity was stronger than the other neoepitopes within the same region (Supplementary Figure 2). C1α1 showed positive staining in the AF region (Supplementary Figure 3), while C1,2C showed positive staining in both AF and NP regions (Supplementary Figure 4). The neoepitope expression profiles in different disc regions (AF, NP, EP) were summarized in Supplementary Tables 1 and 2.

### 3.2 IHC staining of NB100 in NP

**FIGURE 5:**
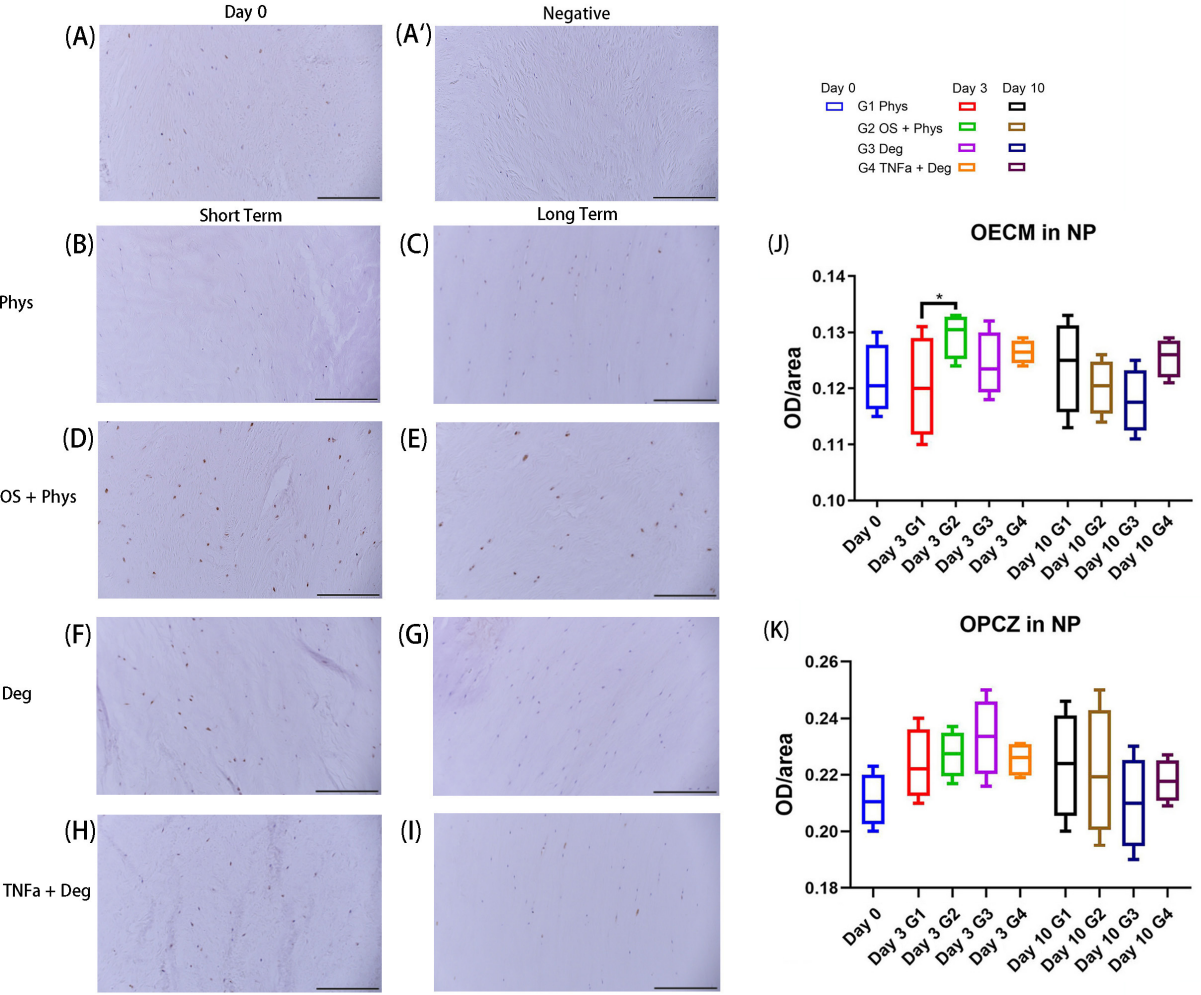
IHC of NB100 in the NP tissue of bovine IVDs subjected to different loading patterns. (A) Representative IHC image from Day 0. (A′) Representative IHC image of negative control from Day 0. (B, D, F, H) Representative IHC images after short‐term culture. (C, E, G, I) Representative IHC images after long‐term culture. (J) OECM in NP. (K) OPCZ in NP. Scale bar 200 μm. *N* = 4, median with interquartile range, *p < 0.05

The staining OECM of NB100 was significantly increased in the OS + Phys group compared with the Phys group at short‐term culture (Figure 5J). No significant difference was found between the other groups at the other time points. For the OPCZ, there was no significant difference between groups at both short term and long term (Figure 5K).

### 3.3 IHC staining of MMPCC in EP and NP

**FIGURE 6:**
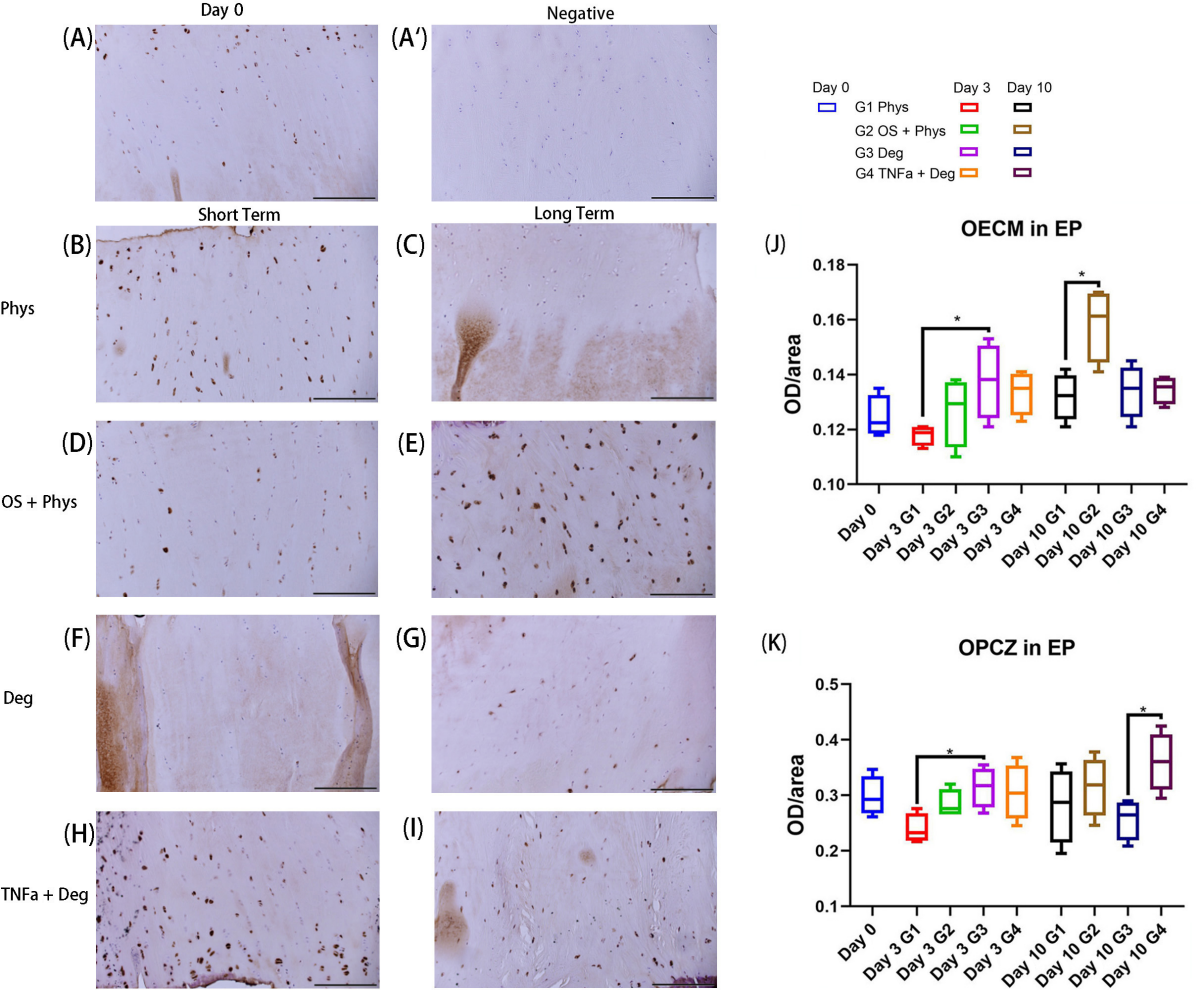
IHC of MMPCC in the EP tissue of bovine IVDs subjected to different loading patterns. (A) Representative IHC image from Day 0. (A′) Representative IHC image of negative control from Day 0. (B, D, F, H) Representative IHC images after short‐term culture. (C, E, G, I) Representative IHC images after long‐term culture. (J) OECM in EP. (K) OPCZ in EP. Scale bar 200 μm. *N* = 4, median with interquartile range, *p < 0.05

The MMPCC IHC staining results in the EP region were shown in Figure 6. On Day 10, the OS + Phys group showed higher MMPCC staining intensity compared with the Phys group in the ECM (Figure 6J, *p* < 0.05). The Deg group showed significantly stronger staining intensity compared with the Phys group in both ECM and PCZ after short‐term culture (Figure 6J,K, *p* < 0.05). For the OECM, Phys group remained low on Day 3 and slightly increased on Day 10. OS + Phys increased markedly after the long‐term culture with loading. After short‐term culture, the OECM of MMPCC increased slightly in the Deg and TNFα + Deg groups and no significant differences were observed between these two groups (Figure 6J). For the OPCZ, Phys group remained low and slightly increased after long‐term culture; TNFα + Deg group was higher than Deg group on Day 10 due to the TNF‐α inflammatory stimuli (Figure 6K).

**FIGURE 7:**
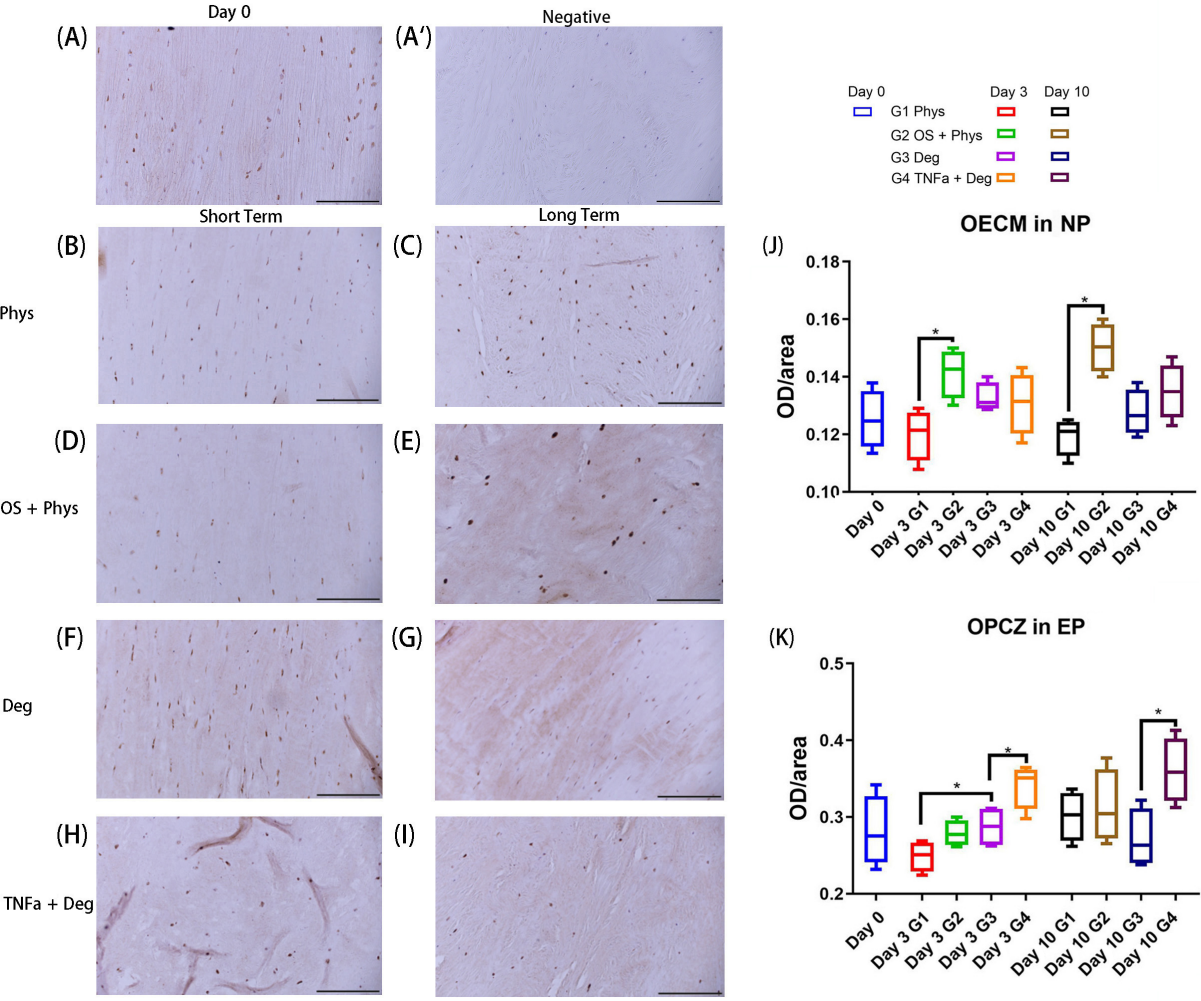
IHC of MMPCC in the NP tissue of bovine IVDs subjected to different loading patterns. (A) Representative IHC image from Day 0. (A′) Representative IHC image of negative control from Day 0. (B, D, F, H) Representative IHC images after short‐term culture. (C, E, G, I) Representative IHC images after long‐term culture. (J) OECM in NP. (K) OPCZ in NP. Scale bar 200 μm. *N* = 4, median with interquartile range, *p < 0.05

The OECM of MMPCC staining in NP tissue of OS + Phys group was significantly higher than the Phys group after both short‐term and long‐term culture (Figure 7J, *p* < 0.05). OECM of Phys group remained low during the whole culture period. OS + Phys was the highest after long‐term culture (Figure 7J). The OPCZ of MMPCC staining in NP tissue of TNFα + Deg group was significantly higher than the Deg group after short‐term and long‐term culture (Figure 7K, *p* < 0.05). The Deg group showed significantly higher OPCZ than Phys group after short‐term culture (Figure 7K, *p* < 0.05). Representative IHC images of MMPCC in EP and NP after long‐term culture from Phys and OS + Phys group are shown in Supplementary Figure 5, showing the specific staining features in the extracellular matrix and pericellular region.

### 3.4 IHC staining of C1α1 in outer AF

**FIGURE 8:**
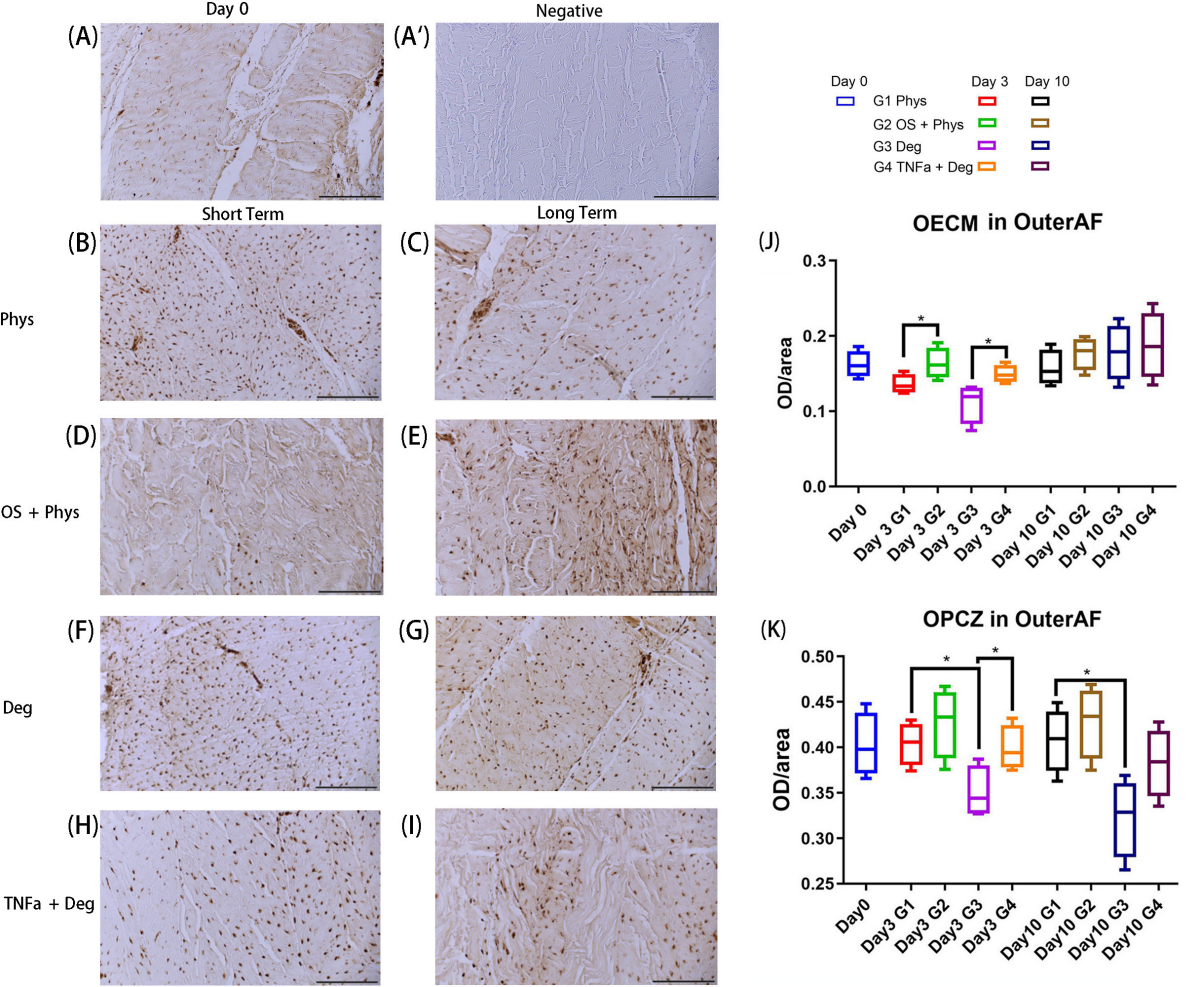
IHC of C1α1 in the outer AF tissue of bovine IVDs subjected to different loading patterns. (A) Representative IHC image from Day 0. (A′) Representative IHC image of negative control from Day 0. (B, D, F, H) Representative IHC image after short‐term culture. (C, E, G, I) Representative IHC image after long‐term culture. (J) OECM in outer AF. (K) OPCZ in outer AF. Scale bar 200 μm. *N* = 4, median with interquartile range, *p < 0.05

The C1α1 staining revealed a significant difference in outer AF between Phys group and Deg group, and between Deg group and TNFα + Deg group in OPCZ after both short‐term and long‐term culture. For the OECM after short‐term culture, OS + Phys group was significantly higher than Phys group; TNFα + Deg group was significantly higher than Deg group (Figure 8J, *p* < 0.05). For the OPCZ, OS + Phys was the highest of the four groups; Deg was significantly lower than Phys (Figure 8K, *p* < 0.05).

### 3.5 IHC staining of C1,2C in outer AF

**FIGURE 9:**
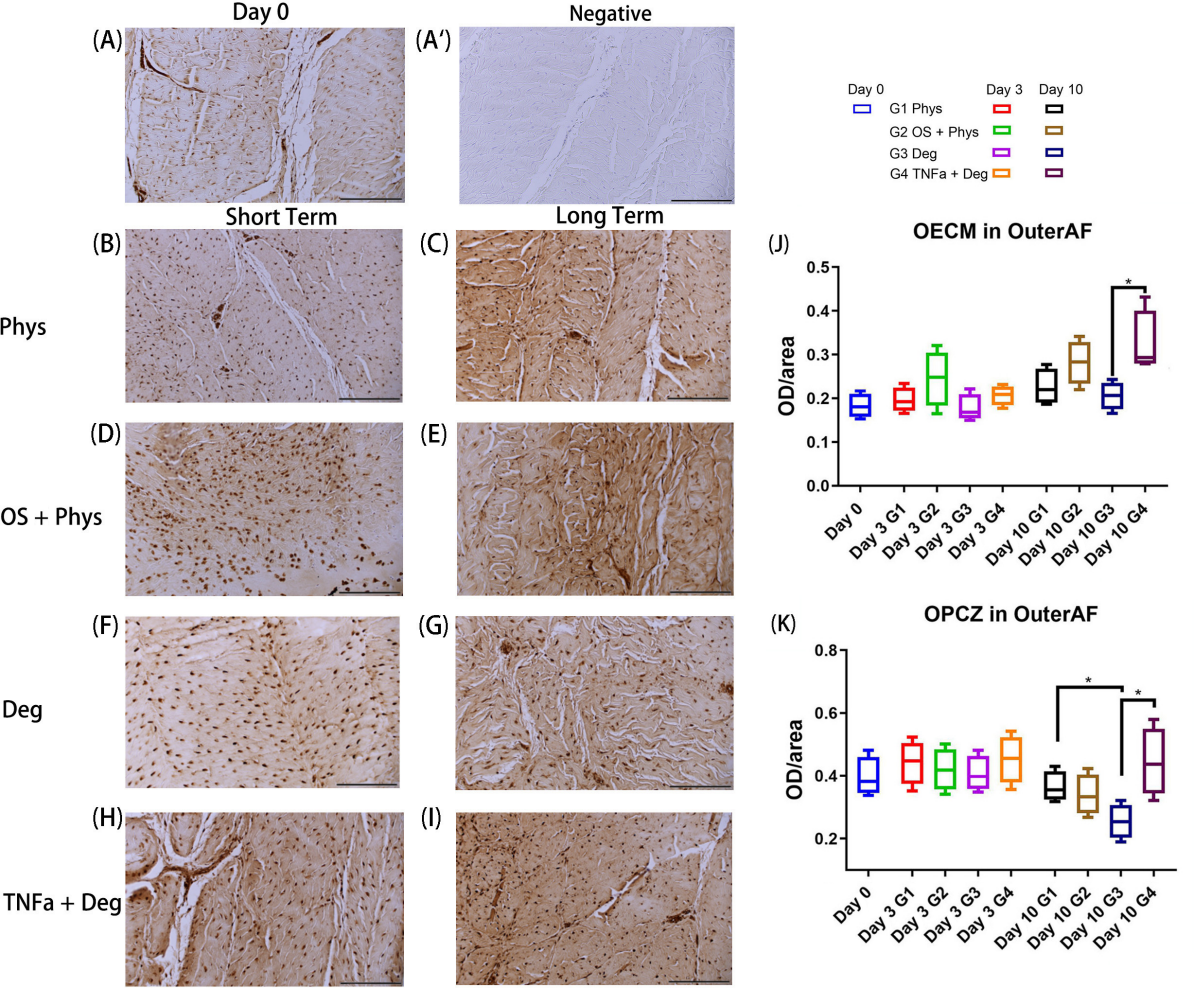
IHC of C1,2C in the outer AF tissue of bovine IVDs subjected to different loading patterns. (A) Representative IHC image from Day 0. (A′) Representative IHC image of negative control from Day 0. (B, D, F, H) Representative IHC image after short‐term culture. (C, E, G, I) Representative IHC image after long‐term culture. (J) OECM in outer AF. (K) OPCZ in outer AF. Scale bar 200 μm. *N* = 4, median with interquartile range, *p < 0.05

For the IHC staining of neoepitope C1,2C, a significant difference was found between Deg and TNFα + Deg groups for all parameters in the outer AF. For the OECM, TNFα + Deg was significantly higher than Deg after long‐term culture (Figure 9J, *p* < 0.05). As for the OPCZ staining after long‐term culture, Deg group was significantly lower than Phys group, TNFα + Deg was significantly higher than Deg (Figure 9K, *p* < 0.05). Representative IHC images of C1,2C in outer AF after long‐term culture from Deg and TNFα + Deg group are shown in Supplementary Figure 5, showing the specific staining features in the extracellular matrix region.

### 3.6 IHC staining of MMPCC and C1,2C in human IVD samples

**FIGURE 10:**
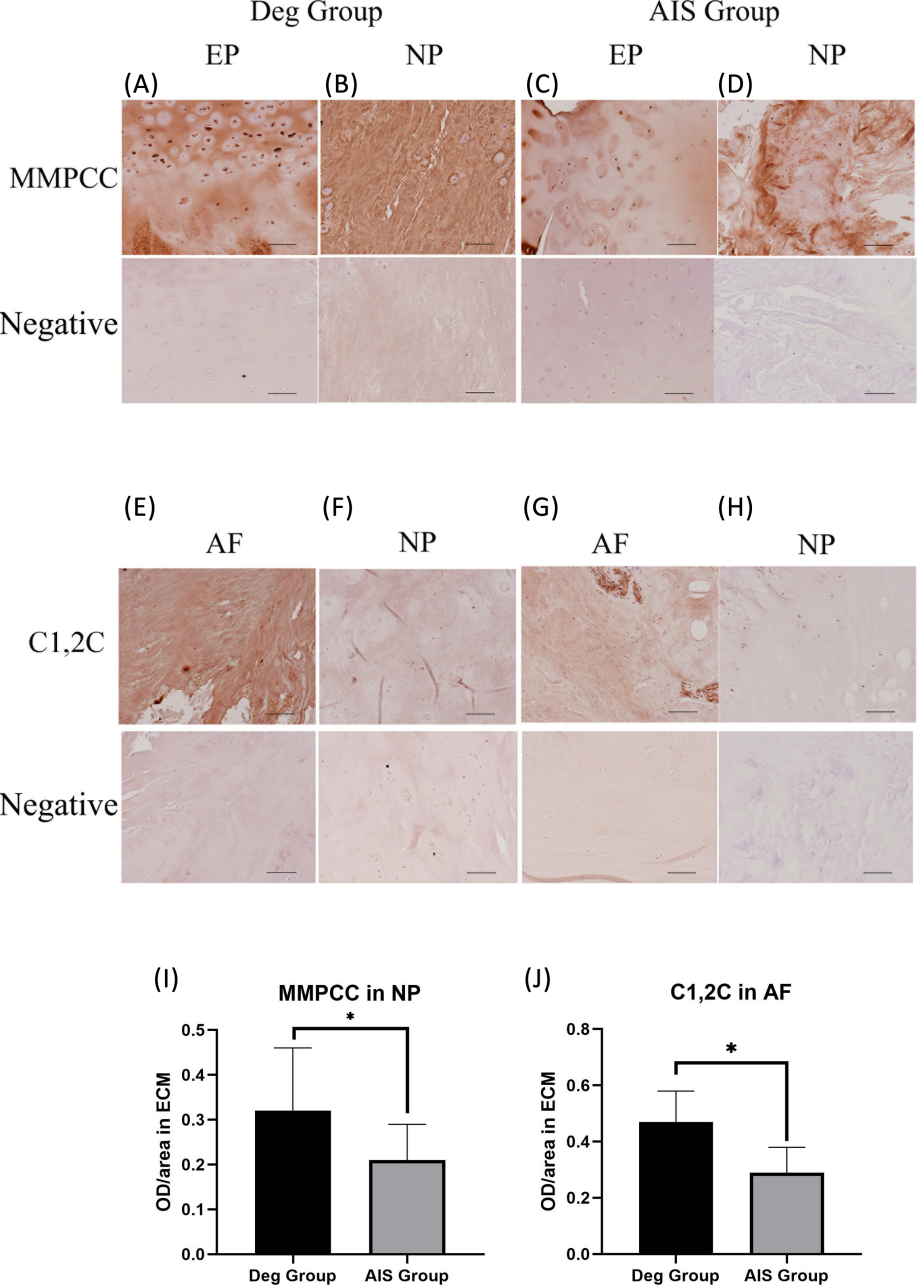
Human IVD samples stained for MMPCC (A, B, C, D) and C1,2C (E, F, G, H). A, B, E, F were from Deg Group and C, D, G, H were from AIS Control Group. (I) OECM of MMPCC IHC in NP tissue. (J) OECM of C1,2C IHC in AF tissue. *N* = 3, median with interquartile range, *p < 0.05, scale bar 100 μm

Human IVD tissues were collected from patients suffering from either lumbar disc herniation (Deg Group) or adolescent idiopathic scoliosis (AIS Control Group). Each tissue was subdivided into three regions of interest which were NP, AF and EP. Representative images of MMPCC and C1,2C IHC are shown in Figure 10. The OECM staining intensity of MMPCC in NP region was observed to be significantly higher in Deg Group compared with the AIS Control Group (*p* < 0.05). Similar finding was observed for C1,2C in the AF region (*p* < 0.05). For the OECM staining of MMPCC in EP and C1,2C in NP, there was no significant difference between groups.

### 3.7 Release of IL‐6, IL‐8 and C1,2C in conditioned medium

**FIGURE 11:**
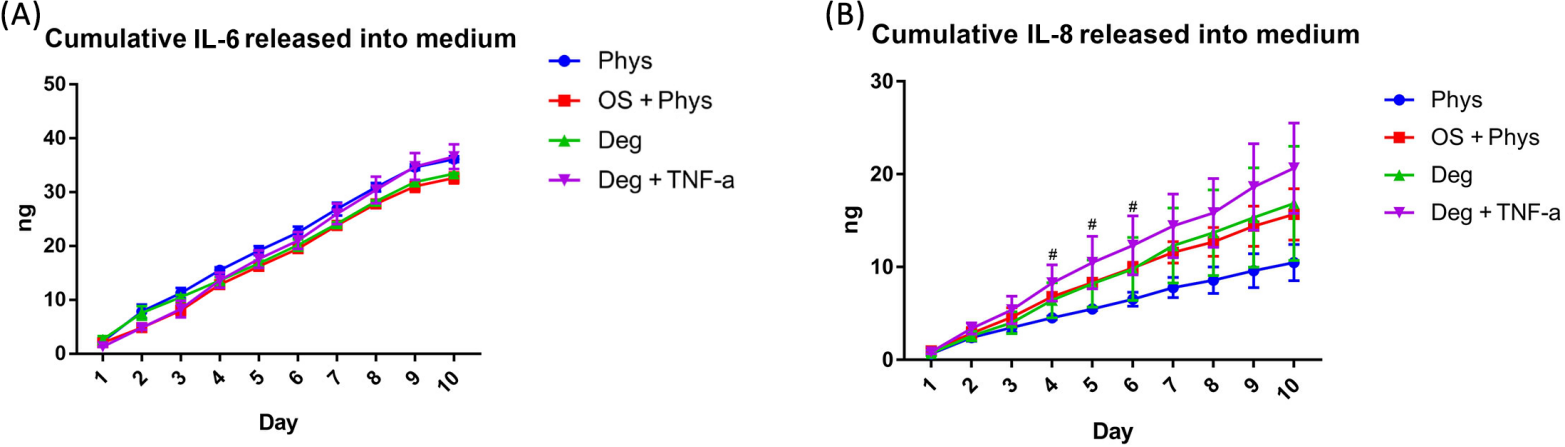
Accumulative IL‐6 (A) and IL‐8 (B) release in the conditioned medium. *N* = 4, ^#^ *p* < 0.05 OS + Phys vs. Phys

Conditioned media were collected daily during the loading period and free swelling period for the measurement of released molecules. There was no statistical difference in cumulative IL‐6 release between groups (Figure 11A). For the cumulative IL‐8 release, a significantly higher release was observed in the OS + Phys group compared with Phys group at Days 4, 5 and 6 (Figure 11B, *p* < 0.05). C1,2C neoepitope was only detected in the Deg + TNF‐α Group at Day 10 after free swelling, with a concentration of 0.045 μg/ml. In all other samples the levels were below the detection limit of the ELISA kit (0.03–10 μg/ml).

## 4 DISCUSSION

The normal morphology and function of ECM are maintained through a balance between anabolism and catabolism. A shift in this equilibrium, toward catabolism, initiates the degradation of the ECM which is closely correlated with IVD degeneration.^43^ Several studies have reported that PG and collagen concentrations in the IVD tissue decreased markedly with aging. Many proteins in the disc undergo degradation during normal turnover with accumulation of fragments. Aggrecan and collagens are the most extensively studied proteins.^7^, ^44^ They are found in cleaved and intact forms within the disc throughout life, and there are numerous proteases capable of cleaving aggrecan and collagens at different sites.^45^ Various causes have been hypothesized to play a role in the pathogenesis of degenerative disc disease, such as adverse loading, trauma, and inflammation. To date there have been no studies describing differences in the topographical distribution of aggrecan and collagen neoepitopes generated by proteolysis in the disc tissue during early degeneration triggered by different IVD degenerative causes. Using the unique IVD organ bioreactor culture system and various degeneration models established in our lab, this study reveals the spatial and temporal expression profile of aggrecan and collagen neoepitopes induced by different IVD degenerative cues.

One strike organ culture model was used to mimic the degenerative situation caused by traumatic injury. In our previous study, the expression of MMPs and ADAMTs was upregulated in this model.^46^ For the MMPCC neoepitope, there was significantly stronger staining in the extracellular matrix (OECM) of EP and NP region after long‐term culture, which is likely due to the enhanced level of MMPs induced by one strike loading in the IVD tissue. Degenerative loading was used to mimic the degenerative situation caused by fatigue loading. Compared with the Phys control group, degenerative loading significantly downregulated C1α1 neoepitope expression in the outer AF region, as indicated by pericellular staining (OPCZ). One possible reason is that the degenerative loading may have facilitated the diffusion of the C1α1 neoepitope from the AF tissue into the adjacent culture medium.

Inflammatory cytokines play an important role during IVD degeneration. Recent research showed that the expression of proinflammatory cytokines, such as TNF‐α is associated with the severity of IVD degeneration in both human and animal models.^32^, ^47^ In our previous study, we compared TNF‐α injection with Phys loading group vs. Phys loading group. No significant changes were found in the expression of inflammatory and catabolic markers. We found that compared with Phy culture condition, Deg culture condition combined with TNF‐α injection reduced COL‐2 gene expression compared with Phy group. Furthermore, a major catabolic enzyme MMP‐1 and inflammatory markers IL‐1β, IL‐6, and IL‐8 showed significant increase under Deg + TNF‐α culture condition compared with Phy group. These results indicate a stronger and additive proinflammatory effect of combined degenerative culture condition and TNF‐α injection, compared with single initiators.^32^ In the current study, we injected TNF‐α into the disc followed degenerative loading to evaluate the contribution of inflammation during the neoepitope production. Compared with the Deg group alone, injection of TNF‐α significantly upregulated the expression of C1,2C neoepitope in the extracellular matrix (OECM) of outer AF region after long‐term culture. The C1,2C staining in the pericellular zone (OPCZ) in the outer AF region were also significantly enhanced by TNF‐α after long‐term culture. These results reflected the activation of proteinases such as MMPs and ADAMTs, which are stimulated by TNF‐α.^48^ These results comparing various bovine IVD degeneration models showed that different neoepitopes were observed to accumulate in specific regions at different time points in response to the specific degenerative stimuli. This neoepitope expression profile reflects the degradation process of IVD ECM and may be used for selection of potential biomarkers in early IVD degeneration.

Human IVD tissue was also utilized to verify the expression profile of neoepitopes, which were identified as promising biomarker based on the results in bovine IVD experiments. Consistent with the bovine IVD organ model results, the OECM of MMPCC in the NP region was significantly higher in the herniated human IVD tissue compared with the AIS control. Previously, human disc tissues of Pfirrmann grade II, III, IV and V (age range 38–82 years) were found to express higher levels of aggrecanase‐generated neoepitopes compared with normal controls which were Pfirrmann grade I.^49^ These results indicate that aggrecan neoepitope levels increase with degeneration Pfirrmann grade in human tissue. Tian et al. used a mouse tail needle puncture model to investigate the expression of aggrecan neoepitope VDIPEN, whose expression was observed from 2 days up to 4 weeks after injury.^50^ Fukuta et al. measured purified aggrecan fragmentation in human degenerated disc tissue by Western blotting and immunohistochemistry with VPGVA antibody, which recognizes the m‐calpain generated neoepitope GVA.^51^ This work found that this neoepitope was expressed in the ECM and correlated with the degree of disc degeneration.^51^ Our bovine IVD organ culture results also showed that aggrecan MMPCC neoepitope was sensitive to traumatic and fatigue loading, as indicated by significant increase and accumulation in ECM of NP and EP regions. These findings suggest that aggrecan neoepitope expression in IVD tissue is elevated during trauma and could be used as a biomarker, revealing early IVD pathological changes.

Collagen and aggrecan degradation and turnover in the IVD matrix have also been measured during aging and degeneration.^52^ Collagenases, including MMP‐1, MMP‐8, MMP‐13, and MMP‐18, predominantly cleave fibrillar collagens. Gelatinases (MMP‐2 and MMP‐9) degrade the denatured collagens, gelatins, and laminin.^53^ MMP expression in normal IVD tissue is low and even devoid. In infant and preadolescent IVDs, MMP‐1, MMP‐2, MMP‐3, and MMP‐9 cannot be detected using immunohistochemistry; however, MMP‐1 and MMP‐3 appear in adult IVDs.^54^ Xu et al. analyzed the relationship between degenerative IVD grade and MMP‐1 expression in disc specimens from patients who had undergone surgery for lumbar disc herniation. The expression of MMP‐1, MMP‐2, and MMP‐14 were found gradually upregulated with increase in degenerative IVD grade.^55^, ^56^ Immunocytochemical localization analysis revealed that MMP‐12 is abundantly present in both the annulus and nucleus regions of human degenerative disc.^57^ Together, these observations suggest that highly expressed MMPs may play an important role in promoting the progression of IVD degeneration. It is well known that loss of aggrecan is an early critical event in the degenerative cascade of IVD tissue. ADAMTS‐4 (aggrecanase‐1) and ADAMTS‐5 (aggrecanase‐2) are currently classified as the major aggrecanases due to their high efficiency in cleaving aggrecan.^58^ Similar to MMPs, several studies have identified significantly increased expression of ADAMTS‐1, ADAMTS‐4, ADAMTS‐5, ADAMTS‐7, ADAMTS‐12, and ADAMTS‐15 in human degenerated IVD tissue compared with non‐degenerated tissue.^59^, ^60^ Our results from bovine IVD organ culture models and human IVD samples add new insight relate to the collagen neoepitope profile induced with different IVD degeneration causes. Li et al. developed a collagen hybridizing peptide (CHP) that hybridizes with the flaw triple helix structure of degraded collagen and found that the CHP fluorescence intensity was correlated with the histological degenerative score.^61^ Another study showed that high‐frequency, low‐amplitude whole‐body vibration in mice increased MMP‐mediated collagen cleavage products in the outer AF compared with the mice without treatment.^62^ Hartman et al. cultured rabbit lumbar IVD segments loaded with cyclic compression and measured the type II collagen degradation fragments in conditioned medium. No difference was found between the loaded and unloaded groups.^63^ Compared with Hartman's study, which only loaded the disc for a short period of time (20 min), our study loaded the disc for longer times and with injection of TNF‐α (TNFα + Deg group). Under these conditions, C1,2C neoepitope was detected in the conditioned medium. These findings suggest that collagen neoepitope was not abundantly released at the early stage of degeneration (loading), but the inflammation factors (TNF‐α) also played an important role for this collagen neoepitope production.

The depletion and fragmentation of ECM proteins due to injury or degeneration can result in release of ECM molecules from the disc into systemic biofluids.^13^, ^64^ In our study, the C1,2C neoepitope was detected in the conditioned medium from the TNFα + Deg group, indicating that the neoepitope can potentially diffuse from the IVD tissue into the surrounding body fluid. This could provide the possibility of a non‐invasive, biochemical means of assessing early disc degeneration.

The limitation is that the sample size of this proof‐of‐concept study is relatively low with the aim of screening various types of neoepitopes in different regions of IVDs within several organ culture models. Further study is warranted with enhanced sample size to confirm the findings from this study with focus on selected neoepitope, tissue region and degeneration organ culture models.

In conclusion, neoepitopes generated by aggrecan and collagen fragmentation were shown to be present in the NP, EP, and AF of degenerated bovine IVD. By analyzing the results from IHC staining, the expression of neoepitopes in the IVD indicated region and time specific expression profiles under different degenerative triggers. Aggrecan MMPCC proved to be a marker of mechanical overload in EP and NP; while collagen C1,2C neoepitope was sensitive to inflammation. Assessment of aggrecan and collagen neoepitopes may be used to evaluate early, severity grade, predict regenerative treatments, and stratify disease phenotypes of IVD degeneration. These, and others not evaluated in this study, neoepitope biomarkers could be used for future diagnostics of IVD disease using specific imaging tools, such as fluorescent molecular tomography^65^ and Raman. These advanced imaging techniques, in combination with other conventional tools (e.g., CT, MRI, clinical data, genetic predisposition) and imagine sensible probes, will be instrumental for clinicians to screen patients at risk of IVD degeneration disease and to better define the proper IVD phenotype, leading to a more appropriate treatment.

## AUTHOR CONTRIBUTIONS

Zhen Li and Mauro Alini designed the study; Shangbin Cui, Wenyue Li, and Graciosa Q. Teixeira performed the experiments; Shangbin Cui analyzed the data; Shangbin Cui drafted the manuscript; Cornelia Neidlinger‐Wilke, Hans‐Joachim Wilke, Lisbet Haglund, Hongwei Ouyang, R. Geoff Richards, Mauro Alini, Sibylle Grad, and Zhen Li provided critical suggestions and discussions throughout the study and revised the manuscript.

## CONFLICT OF INTEREST

The authors declare no conflicts of interest.

## Supporting information


**Appendix S1** Supporting Information
