# Supplementary material for: Neoepitope fragments as biomarkers for different phenotypes of intervertebral disc degeneration
Source: JOR Spine. 2022 Jul 6;5(3):e1215. doi: 10.1002/jsp2.1215 (PMC9520770; doi:10.1002/jsp2.1215)
Supplement: Supplementary file 1 — Appendix S1 Supporting Information [file JSP2-5-e1215-s001.docx]

**Immunohistochemistry protocol for IVD neoepitope**

**Reagents**

1x PBS (for 1L)

0.32 g Sodium dihydrogen phosphate monohydrate NaH_2_PO_4_.H_2_O (Fluka # 71504)

1.42 g di-Sodium hydrogen phosphate dihydrate Na_2_HPO_4_.2H_2_O (Fluka # 71645)

9 g Sodium Chloride NaCl (Fluka # 71380)

1 L Milli-q H_2_O

PBS-T

1L 1x PBS

1 mL Tween 20 (Sigma # P1379)

ABC-complex, Vectastain Elite ABC Kit (Vector Laboratories #PK-6100), 160 µL / slide

1 mL

960 µL PBS-T

20 µL ABC-A

20 µL ABC-B

**Procedures**

1. **deparaffinize sections and bring to dH_2_O**

on hot plate 3 min <60°C

turn on the hot plate at "7", watch the temperature almost 60°C, then turn it off and place the sections on the hot plate for 2 minutes.

2 x xylene (4 minutes each), use clean fresh xylene, lift rack in xylene up and down to remove paraffin (the xylene and 100% ethanol should go to the yellow tank, other ethanol could go to the sink, wear thick blue gloves. )

2 x 100% ethanol (2 minutes each), if there is still paraffin, will see white things in ethanol,

96% ethanol (2 min), 70% ethanol (2 min), 50% ethanol (2 min), dH_2_O

series ethanol to remove xylene, because xylene does not mix with H_2_O, but mix with ethanol

1. **Methanol / H_2_O_2_ 30 min, block endogenous peroxidase**

250 mL of 100% Methanol + 2.5 mL of 30% H_2_O_2_ hydrogen peroxide (prepare in hood, use glass pipette, if in touch with skin, clean well immediately) 250 mL / glass rack

1. Remove slides from methanol, let dry, and draw circle (2-3 circles) around sections with Dako pen (Dako # S2002), let Dako dry completely
2. Block with goat serum, 1:20 dilution in PBS-T, 1 hour at room temperature in a moist chamber

Normal Goat Serum: Vector Laboratories #S-1000

200 µL / slide, mix goat serum and PBS-T in bottle, gentle vortex

Tip off PBS-T on paper towel and add goat serum with pipette. Do 5-7 slides at one time, leave the other slides in PBS-T, so they do not dry out.

1. Tip off goat serum (do not rinse or wash slides, do 5-7 slides at one time) and add primary antibody + 1:200 goat serum, 200 µL / slide, 4°C overnight

Negative controls receive PBS-T+1:200 goat serum

1. Wash in PBS-T, 3x, 5 min each (tip off 1^st^ Ab and put slide in PBS-T one by one)
2. Secondary antibody, 200 µL / slides, dilute 1:200 with PBS-T, 30 min at RT.

Tip off PBS-T on paper towel and add 2^nd^ Ab with pipette. Do 5-7 slides at one time, leave the other slides in PBS-T, so they do not dry out. In the meantime, prepare the ABC-complex (160 µL / slide) which should be prepared at least 30 minutes before using, and keep at 4°C.

1. Wash in PBS-T, 3x, 5 min each (tip off 2nd Ab and put slide in PBS-T one by one)
2. ABC-complex, 160 µL / slide, 30 min at RT

Tip off PBS-T on paper towel and add ABC with pipette. Do 5-7 slides at one time, leave the other slides in PBS-T, so they do not dry out.

1. Wash in PBS-T, 3x, 5 min each (tip off ABC and put slide in PBS-T one by one)
2. DAB solution, 160 µL / slide, 5 min in the dark,

ImmPACT DAB 1 mL Buffer + 1 drop, vortex

Do 10 slides at one time, turn off light, prepare paper towel for dipping off DAB (carcinogenic)and rack of dH_2_O.

Wear two pairs of gloves, the left hand keep clean with the shelf (ready to left it up when put the slide in), the right hand can contact the slide .

Because the DAB is poisoness, you have to put the paper towel in the white tray.

1. Stop reaction by rinsing in dH_2_O, 10 min, change 3x in between
2. Counterstain with Mayer's Hematoxylin (Fluka # 51275) 15 seconds only, otherwise too dark. Filter before use, pour back to bottle after use without filtering.
3. Blue in tap water (put slides in glass rack, do not run water directly on slides), 5 min, then rinse with dH_2_O
4. Dehydrate in ascending ethanol, 2 min each: 50%, 70%, 96%, 100%, 100% ethanol
5. Clean in xylene, change to fresh xylene, and mount with Eukitt (Fluka # 03989)

Clean eukitt container with ethanol on top of the organic waste tank before using. Put it back in xylene container after using.

Xylene for cleaning is for the container.

Xylene1 is for the first time from the ethanol to the xylene

Xylene2 is the last step of xylene clean, you can leave the slides in the box until it can be used.

The eukitt can be put back into the bottle where it taken from.

Leave slides on heating plate for 1 hour, then put it into the bookbox


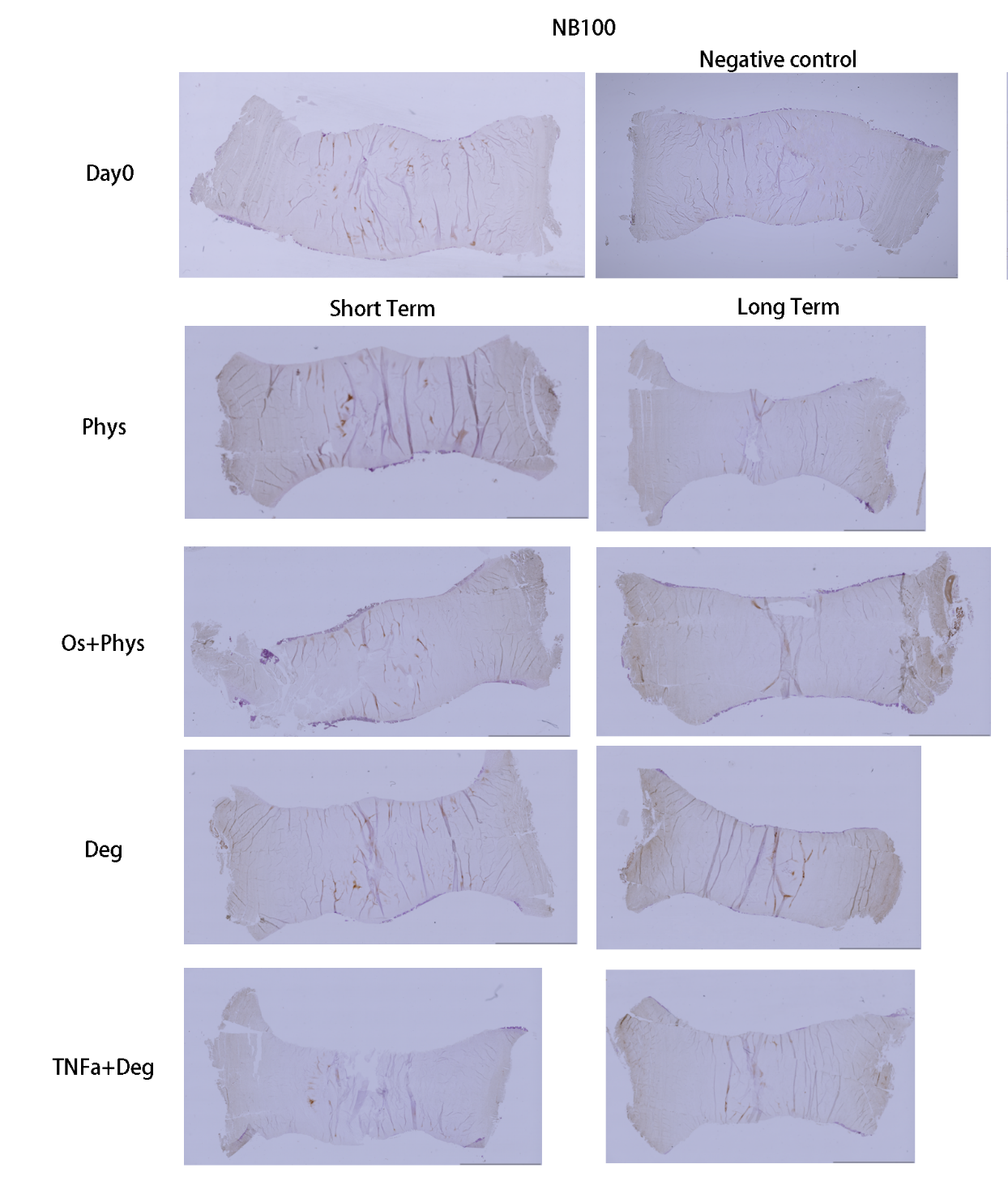


**Supplementary Figure 1.** IHC of Aggrecanase Cleaved C-terminus Aggrecan Neoepitope (NB100). Scale bar 5 mm.


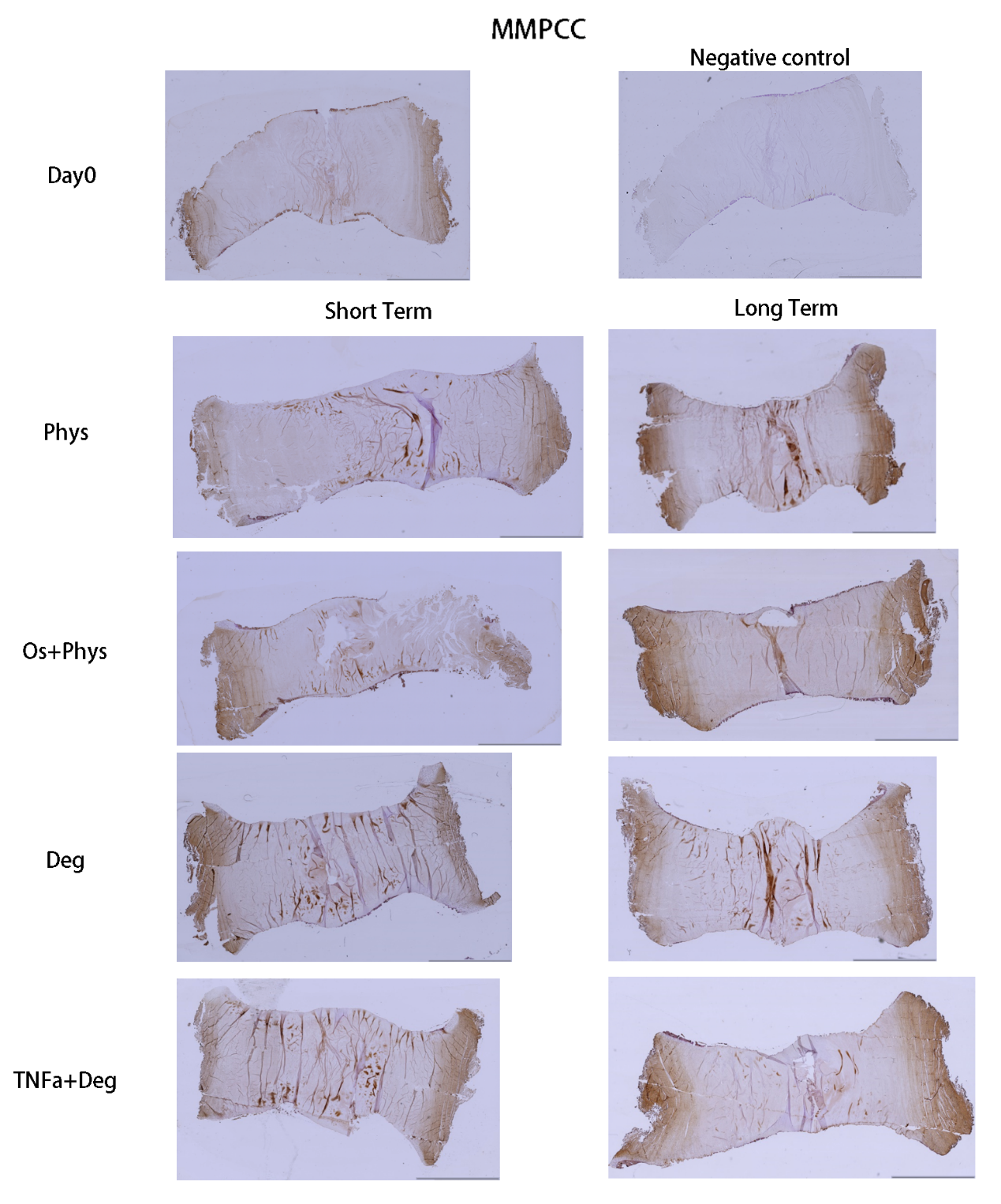


**Supplementary Figure 2.** IHC of MMP Cleaved C-terminus Aggrecan Neoepitope (MMPCC). Scale bar 5 mm.


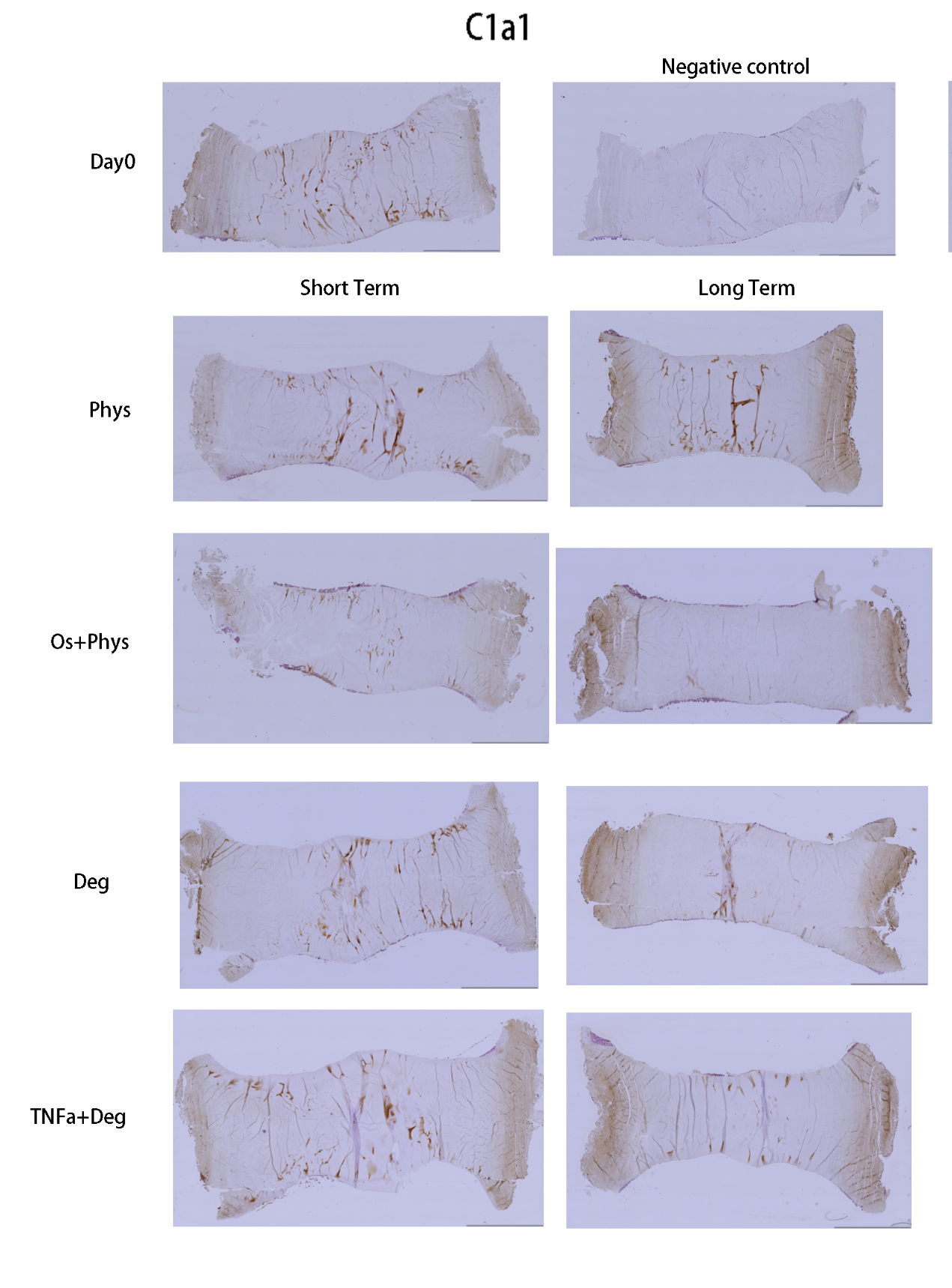


**Supplementary Figure 3.** IHC of Collagen Type 1α1, 1/4 Fragment Neoepitope (C1α1). Scale bar 5 mm.


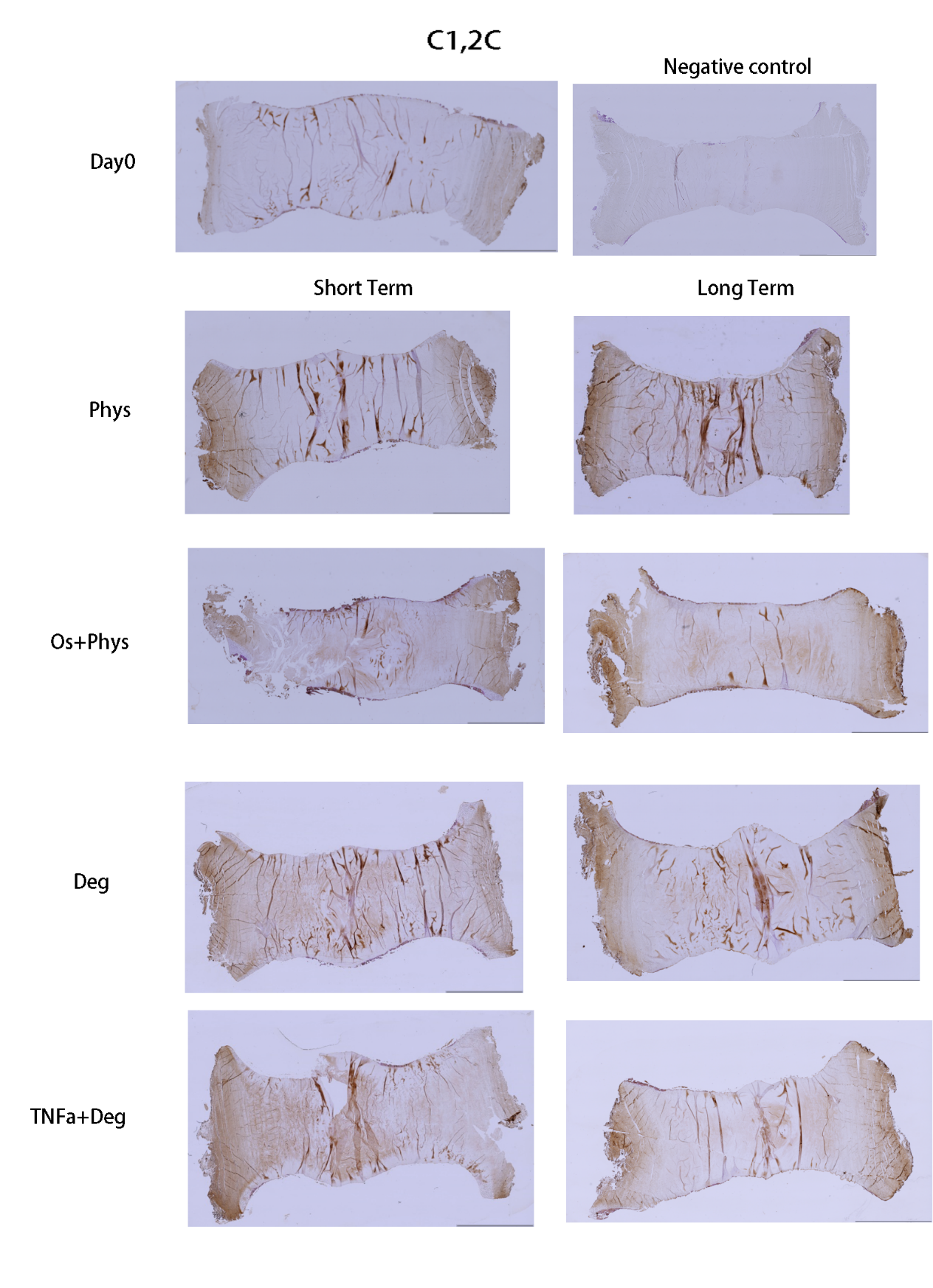


**Supplementary Figure 4.** IHC of Collagenase (MMPs) Cleaved Type Ⅰ and Ⅱ Collagen Neoepitope (C1,2C). Scale bar 5 mm.


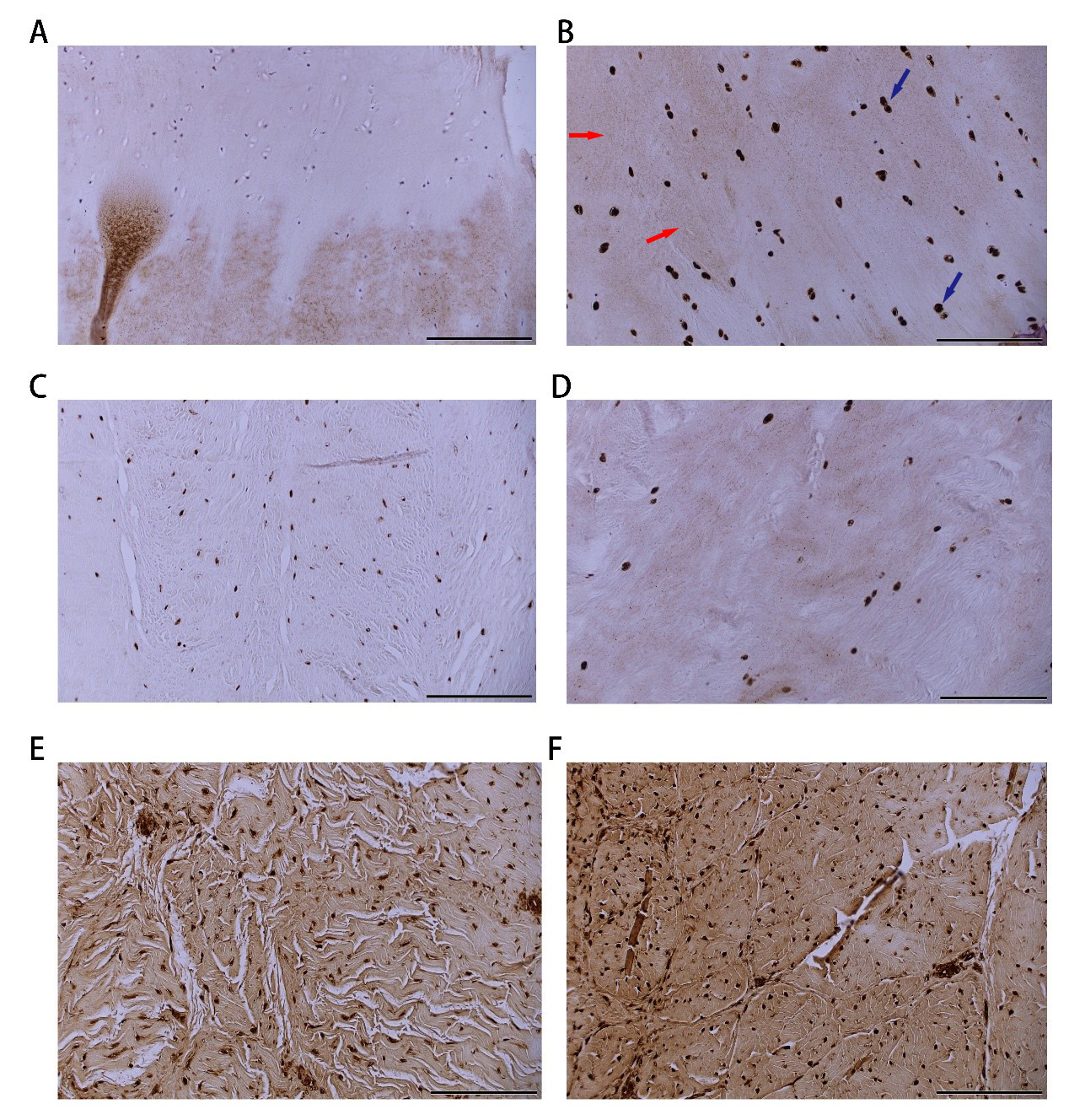


**Supplementary Figure 5.** Representative IHC images of MMPCC in EP after long-term culture from Phys (A) and OS+Phys (B) group, showing stronger staining intensity in the extracellular matrix and pericellular region in OS+Phys group compared with Phys group. The red arrow indicates the OECM staining, and the blue arrow indicates the OPCZ staining. Representative IHC images of MMPCC in NP after long-term culture from Phys (C) and OS+Phys (D) group, showing stronger staining intensity in the extracellular matrix and pericellular region in OS+Phys group compared with Phys group. Representative IHC images of C1,2C in outer AF after long-term culture from Deg (E) and Deg+TNFα (F) group, showing stronger staining intensity in the extracellular matrix region in Deg+TNFα group compared with Deg group. Scale bar 200 μm.

**Supplementary Table 1:** Neoepitope expression in ECM of different disc region

| Neoepitope | IVD degeneration triggers | | |
| --- | --- | --- | --- |
|  | Traumatic injury | Fatigue loading | Inflammation |
|  | Group 2 | Group 3 | Group 4 |
| NB100 | NP(S), EP(L) | EP(S) |  |
| MMPCC | EP(S)/NP(S, L) | EP(S) |  |
| C1α1 | AF(S) |  | AF(S) |
| C1,2C |  |  | AF(L) |

“S” stands for short term where disc was cultured for 3 days. “L” stands for long term where disc was cultured for 10 days.

**Supplementary Table 2:** Neoepitope expression in PCZ of different disc region

| Neoepitope | IVD degeneration triggers | | |
| --- | --- | --- | --- |
|  | Traumatic injury | Fatigue loading | Inflammation |
|  | Group 2 | Group 3 | Group 4 |
| NB100 |  |  |  |
| MMPCC |  | NP(S), EP(S) | NP(S, L), EP(L) |
| C1α1 |  | AF(S, L) | AF(S) |
| C1,2C |  | AF(L) | AF(L) |

“S” stands for short term where disc was cultured for 3 days. “L” stands for long term where disc was cultured for 10 days.
